# Evaluation of the effect of crocin on prevention of atrial fibrillation after coronary artery bypass grafting or heart valve replacement: A double-blind, randomized, placebo-controlled trial

**DOI:** 10.22038/AJP.2022.20908

**Published:** 2022

**Authors:** Seyyed Hamed Hashemi Shahri, Ghasem Soltani, Mohammad Abbasi Teshnizi, Aliasghar Moeinipour, Mohammad Tayyebi, Reza Javidi Dasht Bayaz, Farshad Abedi, Vahid Ghavami, Shahram Amini, Amir Hooshang Mohammadpour

**Affiliations:** 1 *Department of Clinical pharmacy, School of Pharmacy, Mashhad University of Medical Sciences, Mashhad, Iran*; 2 *Department of Anesthesia, School of Medicine, Mashhad University of Medical Sciences, Mashhad, Iran *; 3 *Cardiac Surgeon, Department of Cardiac Surgery, Mashhad University of Medical Sciences, Mashhad, Iran*; 4 *Vascular and Endovascular Surgery Research Center, Faculty of Medicine, Mashhad University of Medical Sciences, Mashhad, Iran*; 5 *Cardiovascular Research Center, Faculty of Medicine, Mashhad University of Medical Sciences, Mashhad, Iran*; 6 *Department of Biostatistics, Social Determinants of Health Research Center, Mashhad University of Medical Sciences, Mashhad, Iran*

**Keywords:** Atrial fibrillation, Coronary artery bypass graft, Heart valve replacement, Crocin

## Abstract

**Objective:**

The aim of this study was to evaluate the effect of crocin on the prevention of atrial fibrillation after coronary artery bypass grafting (CABG) and heart valve replacement.

**Materials and Methods:**

100 patients who were scheduled for CABG or heart valve replacement surgeries were randomly assigned into two groups of treatment and placebo. In the treatment group, patients received crocin tablets from three days prior to surgery and on the first three postoperative days (for a total of six days). During the first three days after surgery, postoperative atrial fibrillation (POAF) was assessed by electrocardiogram monitoring. Prooxidant–antioxidant balance (PAB) and c-reactive protein (CRP) levels were also assessed.

**Results:**

POAF developed in 7 patients in the treatment group versus 18 patients in the control (p=0.02). PAB levels were significantly lower in the crocin group (p<0.001), while differences in CRP levels were insignificant (p=0.39).

**Conclusion:**

It seems that prophylactic use of crocin is effective in reducing the incidence of POAF in patients undergoing heart surgeries.

## Introduction

Atrial fibrillation (AF) is the most common type of arrhythmia which is defined as an irregular and rapid beating of the atrial chambers, potentially leading to blood clots in the heart. AF is common after open heart surgery and is experienced by 25 to 40% of patients undergoing coronary artery bypass grafting (CABG) or heart valve replacement (Zipes et al., 2019 ▶). Its prevalence is higher in the first 48 hr after surgery and decreases within 4 to 7 days after surgery. Postoperative atrial fibrillation (POAF) is associated with consequences such as stroke, cognitive changes, renal problems, prolonged hospital stay, readmission, and death during recovery time after surgery (St-Onge et al., 2018 ▶).

Currently, the exact mechanism of POAF is unknown but multifactorial models including clinical and operational predisposing factors are suggested (Royster et al., 2017 ▶; Thijs et al., 2017 ▶). Importantly, several studies have linked inflammation and oxidative stress to predispose AF in the literature and inflammation and oxidative stress are considered as independent predictors of POAF (Li et al., 2010 ▶; Zakkar et al., 2015 ▶). Subsequently, several studies have demonstrated that medicines with anti-inflammatory and antioxidant characteristics are able to prevent POAF (Bockeria et al., 2017 ▶; Nomani et al., 2021 ▶; Rodrigo et al., 2013 ▶). 

Crocin is a natural water-soluble carotenoid isolated from the saffron (*Crocus sativus*) and is responsible for producing the color of saffron. Chemically, it is a diester from the dicarboxylic acid crocetin and the disaccharide gentiobiose (Huang et al., 2019 ▶; Alavizadeh and Hosseinzadeh, 2014 ▶). In addition to its anti-hypertensive, anti-depressant, anti-atherosclerotic and nephroprotective properties, crocin has significant antioxidant and anti-inflammatory effects. It scavenges reactive oxygen species (ROS) and free radicals, especially superoxide anions (Korani et al., 2019 ▶; Hashemzaei et al., 2020 ▶; Kocaman et al., 2019 ▶; Pakfetrat et al., 2019 ▶).

To date, no studies have been performed on the effects of crocin in prevention of AF after heart surgery. The aim of our study was to investigate the probable role of crocin in reducing and prevention of POAF after CABG and heart valve replacement possibly through anti-inflammatory and antioxidant properties.

## Materials and Methods

**Study design**

This prospective randomized, double-blind, placebo-controlled study was conducted at Imam Reza Hospital, Mashhad, Iran between January 2021 to June 2021. The study was registered at the Iranian Registry of Clinical Trials (registration # IRCT20120520009801N4) and approved by internal review board and the Ethics Committee of Mashhad University of Medical Sciences (IR.MUMS.REC.1399.469).

**Patient selection**

After checking the inclusion and exclusion criteria, 100 patients of both sexes were enrolled in the study. All patients were informed and they gave their written consent. The inclusion criteria were as follows: all adult patients undergoing elective off-pump CABG or heart valve replacement surgery, age between 18 to 70 years old and taking an angiotensin-converting enzyme (ACE) inhibitor/angiotensin receptor blocker (ARB), statins, aspirin and β- blockers before and after surgery for CABG patients. Exclusion criteria were as follows: having a pacemaker, chronic kidney disease (GFR ≥45 ml/min/1.73m^2^), severe hepatic insufficiency (increased liver enzymes more than three times from the upper normal limit), sensitivity to crocin or saffron (assessed by the questionnaire provided in the case record form), history of taking anti-inflammatory drugs other than aspirin or antioxidants during the two weeks before the surgery (assessed based on comprehensive drug history in the case record form), thyroid dysfunction, having an AF rhythm from beginning, or history of any heart surgery, pregnancy, or lactation.

**Randomization and interventions**

Patients were randomly assigned in two groups via permuted block method with size 4 in each block using www.randomization.com. Patients and investigators were all blinded to the treatment allocation and all the patients underwent preoperative echocardiography, electrocardiography (ECG), and selective coronary angiography. Baseline information including demographic characteristics, risk factors, medication history, echocardiographic examination and laboratory measurements were collected. Additionally, the total length of intensive care unit (ICU) stay, the amount of blood received during surgery, the duration of pump perfusion and duration of aortic cross-clamp were recorded.

Tablets containing 15 mg crocin with the brand name of Krocina^®^ were prescribed in this study.

Patients in the treatment group received 15 mg crocin tablets twice daily from three days prior to surgery and on the first three postoperative days. Individuals in the control group received equal number of placebo tablets which had exactly similar appearance as the crocin tablet. 

Both groups had the same surgical premedication and anesthesia protocol. Anesthesia was induced with midazolam, sufentanil, and pancuronium bromide and was maintained with sufentanil, midazolam and propofol. CABG patients continued to take previously prescribed drugs, including ACE inhibitor/ARB, statins, aspirin and β- blockers during the study. 

**Measurements**

After the surgery, the patients were transferred to intensive coronary care unit (ICCU) where continuous ECG monitoring was performed. According to the study protocol, ECG monitoring was carried out continuously during the first 72 hr after the surgery. Patients' ECGs were continuously recorded by the central ECG monitoring system and then read by the resident of cardiology every 12 hr The incidence of POAF and other supraventricular arrhythmias including atrial flutter, atrioventricular nodal reentrant tachycardia (AVNRT) and atrioventricular reentrant tachycardia (AVRT), as well as ventricular arrhythmias including premature ventricular contraction (PVC), ventricular tachycardia (VT) and ventricular fibrillation (VF) were assessed during this period. POAF was defined as the occurrence of at least one episode of AF (with or without symptoms) lasting more than five min. 

Echocardiographic examination was used for the assessment of left and right ventricular systolic and diastolic dysfunction, and for determination of left atrial (LA) or right atrial (RA) enlargement. 

Left ventricular (LV) systolic dysfunction is defined as heart failure (HF) with reduced left ventricular ejection fraction (EF≤40%). In LV diastolic dysfunction, ejection fraction is preserved (EF≥50%). Right ventricular (RV) systolic and diastolic dysfunction has the same definitions but about the ejection fractions of RV (McDonagh et al., 2021 ▶). LA volume index of greater than 34 ml/m^2^ in men and 29 ml/m^2 ^in women was considered LA enlargement. RA volume index cut off values of 23 ml/m^2^ and 19 ml/m^2^ were also considered RA enlargement in men and women, respectively (Baysan et al., 2017 ▶). 

Blood samples were collected before and on the third day after the surgery to assess pro-oxidant-antioxidant balance (PAB) and C-reactive protein (CRP) levels as biomarkers of oxidative stress and inflammation process, respectively. PAB is used routinely as a fast, easy, and cost-effective way in a single assay (Korkmaz et al., 2013 ▶; Taghizadeh et al., 2021 ▶). Some studies have predicted that a high level of PAB is related to increased ROS production that leads to oxidation and cell damage (Ghazizadeh et al., 2020 ▶). Moreover, CRP levels in the plasma is routinely measured as an indicator of inflammation. CRP is an acute-phase reactant protein of hepatic origin; its circulating concentrations increases in response to acute and chronic inflammatory conditions (Mantovani et al., 2008 ▶; Pepys and Hirschfield, 2003 ▶). Serum CRP was assayed using ELISA and enzyme-linked immunoassay kits (LDN, Nordhorn, Germany). To assess PAB, serum samples were analyzed for concentrations of H_2_O_2_ levels via using the method described in the literature (Alamdari et al., 2008 ▶).

**Statistical analysis**

Based on the previous trials, AF during hospitalization has been reported in around 35% of the patients undergoing CABG or heart valve replacement surgery (Zipes et al., 2019 ▶). At a power of 80% and 5% type I error, the sample size needed to detect an absolute difference of 25% in the rate of POAF between the two study groups was 40 subjects in each group. Also, Kolmogorov–Smirnov test was used in order to ensure the normality of distribution. Data describe mean values±standard deviation (SD) for continues variables and percentages for categorical variables. Chi-square test or the Fisher exact test was used to compare categorical variables and the mean variables were compared between the two groups using an independent t-test or the Mann- Whitney U test. We employed multiple logistic regression to adjust the effect of variables with heterogeneous distribution between the study groups. All statistical analyses were performed using the Statistical Package for Social Sciences (SPSS) version 20 (SPSS Inc., Chicago, Ill., USA). A p<0.05 was considered statistically significant.

## Results

**Patient characteristics**

**Figure 1:**
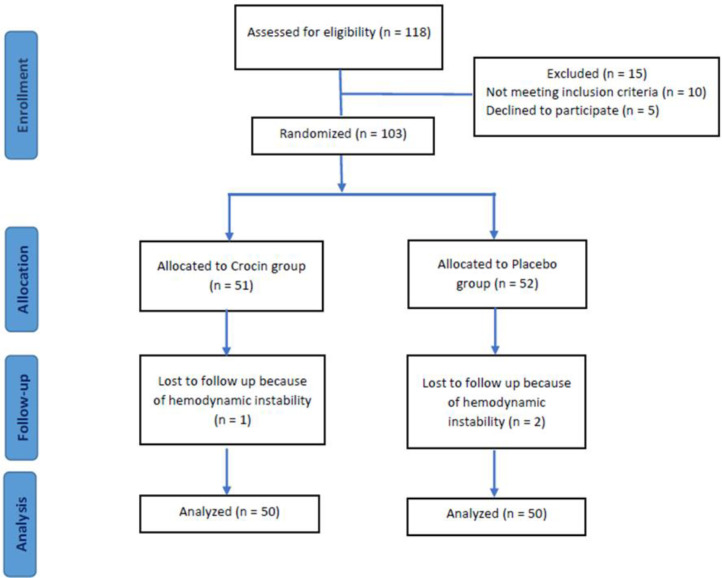
The CONSORT flow diagram of the study

A total of 100 patients (crocin group, n=50 and placebo group, n=50) with the mean age of 56.09±10.01 years old were included in our study. The CONSORT flow diagram is shown in Figure 1. The baseline demographic, clinical, laboratory, and echocardiography parameters of the two groups are presented in Table 1. There were no statistically significant differences between the two groups (p>0.05), except for gender (p=0.03) and systolic blood pressure (p=0.04). But we were confident that this would not affect the study results and supposed that the groups were relatively homogeneous and their further comparison was eligible. 

**ECG findings**

Along the intervention, ECG monitoring during 72 hr after the surgery revealed that AF occurred in 7 patients (14%) in the crocin group in comparison with 18 patients (36%) in the placebo group (p=0.02). So, the incidence of POAF was significantly lower in the crocin group. Ventricular arrhythmias were observed in 48 patients (96%) in the crocin group and in 50 patients (100%) in the placebo group (p=0.49). Furthermore, other supraventricular arrhythmias (except AF) were diagnosed in 10 patients (20%) in the crocin group and in 18 patients (36%) in the placebo group (p=0.11). Accordingly, no significant differences were observed between the groups in the incidence of ventricular or supraventricular arrhythmias except AF (Table 2).

Subgroup analysis was performed between patients who underwent CABG (n=80) and patients who underwent heart valve replacement surgery (n=20). The incidence of POAF was 23.75% (19 patients) in the CABG subgroup while 30% (6 patients) in the valve replacement subgroup (p=0.57). About ventricular arrhythmias, the incidence was 97.5% (78 patients) in the CABG subgroup and 100% (20 patients) in the valve replacement subgroup (p=1). Furthermore, supraventricular arrhythmias (except AF) tended to be lower in the CABG subgroup; 23.75% (19 patients) versus 45% (9 patients) in the valve replacement subgroup (p=0.09). Consequently, there were no statistically significant differences in any type of post-operative arrhythmias between the two subgroups (Table 3). 

**Effects on PAB and CRP levels**

As shown in Table 4, there was no significant difference in serum levels of PAB between the two groups before intervention (p=0.37). But after the intervention, PAB levels were significantly lower in the crocin group than the placebo group (p<0.001). Also, the differences in PAB levels between the crocin and placebo groups were significant before and after the intervention (p<0.001). Interestingly, PAB levels decreased significantly in the crocin group (111.04±23.18 vs. 71.20±15.47, p<0.001) and increased significantly in the placebo group (114.08±20.74 vs. 121.42±23.25, p<0.001) after intervention. Same measurements are presented for CRP levels in Table 5. There was no significant difference in CRP levels between the two groups before the intervention (p=0.43), as well as after the intervention (p=0.22). The differences in the serum levels of CRP between the control and treatment groups were also insignificant before and after the intervention (p=0.39). Furthermore, CRP levels increased significantly in both crocin (3.90±8.18 vs. 117.11±45.32, p<0.001) and placebo (2.82±4.44 vs. 132.26±74.28, p<0.001) groups after the intervention.

**Table 1:** Comparisons of the patient baseline characteristics between the two groups

**p-value**	**Placebo (n=50)**	**Crocin (n=50)**			
0.84	56.00±9.89	56.18±10.22	**Age**		
0.64	25.25±4.11	24.89±3.72	**BMI**		
0.03	26 (52.0%)	37 (74.0%)	**Male gender**		
0.94	40 (80.0%)	40 (80.0%)	CABG		**Operation type**
	4 (8.0%)	3 (6.0%)	MVR	Valve Replacement	
	2 (4.0%)	1 (2.0%)	TVR		
	2 (4.0%)	4 (8.0%)	AVR		
	2 (4.0%)	2 (4.0%)	AVR + MVR		
1.00	9 (18.0%)	9 (18.0%)	**Cigarette smoking**		
0.52	4 (8.0%)	7 (14.0%)	DM		**PMH**
0.15	15 (30.0%)	8 (16.0%)	HTN		
0.52	18 (36.0%)	14 (28.0%)	MI		
0.78	43 (86%)	41 (82%)	ACEI/ARB		**DH**
1.00	47 (94%)	47 (94%)	ASA		
0.79	42 (84%)	40 (80%)	Statins		
0.41	44 (88%)	40 (80%)	β-Blocker		
0.17	11 (22%)	5 (10%)	CCB		
0.21	11	12	LVSD		**ECHO**
0.90	15	13	LVDD		
0.24	6	2	RVSD		
0.55	10	12	LA enlargement		
0.34	7	3	RA enlargement		
0.04	134.98±20.59	128.44±17.43	**pre_SBP**		
0.09	82.40±13.84	78.14±11.31	**pre_DBP**		
0.31	81.24±12.45	77.88±11.90	**pre_PR**		
0.89	0.16±0.03	0.16±0.02	**pre_PR interval**		
0.11	0.08±0.02	0.08±0.02	**pre_QRS interval**		
0.73	0.38±0.02	0.38±0.02	**pre_QT interval**		
0.86	35.77±14.11	35.63±17.49	**pre_PAP**		
0.63	46.72±13.54	49.44±10.14	**pre_LVEF**		
0.46	0.93±0.15	0.91±0.18	**pre_Cr**		
0.37	114.08±20.74	111.04±23.18	**pre_PAB**		
0.43	2.82±4.44	3.90±8.18	**pre_CRP**		
0.07	8368.40±2150.62	7665.40±1819.28	**pre_WBC**		
0.89	150.11±96.69	143.18±54.79	**pre_BS**		

ACEI, angiotensin-converting enzyme inhibitor; ARB, angiotensin receptor blocker; AVR, aortic valve replacement; BMI, body mass index; BS, blood sugar; CABG, coronary artery bypass grafting; CCB, calcium channel blocker, DBP, diastolic blood pressure; CRP, C-reactive protein; DM, diabetes mellitus; HTN, hypertension; LA, left atrial; LVDD, left ventricular diastolic dysfunction; LVEF, left ventricular ejection fraction; LVSD, left ventricular systolic dysfunction; MI, myocardial infarction; MVR, mitral valve replacement; PAB, prooxidant–antioxidant balance; PAP, pulmonary arterial pressure; RA, right atrial; RVSD, right ventricular systolic dysfunction; SBP, systolic blood pressure; SrCr, serum creatinine; TVR, tricuspid valve replacement; WBC, white blood cell

**Table 2:** Comparison of the frequencies of cardiac arrhythmias between the treatment and placebo groups

	**Crocin**	**Placebo**	**p-value**
**Atrial fibrillation**	7 (14%)	18 (36%)	0.02
**Ventricular arrhythmia**	48 (96%)	50 (100%)	0.49
**Supraventricular arrhythmia (except atrial fibrillation)**	10 (20%)	18 (36%)	0.11

**Table 3:** Subgroup analysis of the arrhythmia incidence according to operation type

	**CABG Subgroup (n=80)**	**Valve replacement subgroup (n=20)**	**p-value**
**Atrial fibrillation**	19 (23.75%)	6 (30%)	0.572
**Ventricular arrhythmias**	78 (97.5%)	20 (100%)	1
**Supraventricular arrhythmia (except atrial fibrillation)**	19 (23.75%)	9 (45%)	0.09

**Table 4:** Prooxidant–antioxidant balance (PAB) results at pre-and post-operation times in the two groups

**PAB**	**Mean±SD**		**Between group**
	**Placebo**	**Crocin**	
**Before intervention**	114.08±20.74	111.04±23.18	Z = -87.07 p = 0.37
**After intervention**	121.42±23.25	71.20±15.47	Z = -8.27 p<0.001
**Diff**	7.34±13.19	-39.83±26.09	Z = -8.43 p<0.001
**Within group**	Z =-5.26 p<0.001	Z = -6.13 p<0.001	

**Miscellaneous findings**

Other results of this study are shown in Table 6. There were significant differences between the crocin and placebo groups in terms of hospital stay duration (p=0.004), the mean of blood transfusions during the operation period and ICU stay (p=0.04), as well as the duration of ICU stay (p=0.01). ECG studies revealed statistically significant differences in PR intervals, one, two and three days after the surgery with p-value of 0.005, 0.03 and 0.03, respectively. QRS interval durations were also significantly different on the third day after the surgery (p=0.005), however, QT interval changes were insignificant. The differences in other intra-postoperative characteristics of the patients between the two groups were not statistically significant.

Finally, in the multivariable logistic regression analysis, it was demonstrated that the only predictor of POAF after CABG surgery was the use of crocin (Table 7). 

**Table 5:** CRP results at pre-and post-operation times in the two groups

**CRP**	**Mean±SD**		**Between group**
	**Placebo**	**Crocin**	
**Before intervention**	2.82±4.44	3.90±8.18	Z = -0.77 P = 0.43
**After intervention**	132.26±74.28	117.11±45.32	T = -1.24 P = 0.22
**Diff**	129.43±74.46	113.21±46.00	Z = -0.85 P = 0.39
**Within group**	Z = -6.15 p<0.001	Z = -6.15 p<0.001	

**Table 6:** Intra-postoperative characteristics of the two groups

	**Crocin**	**Placebo**	**p-value**
**Day1_ PR interval**	0.17±0.02	0.15±0.02	0.005
**Day2_ PR interval**	0.16±0.02	0.15±0.02	0.03
**Day3_ PR interval**	0.18±0.02	0.16±0.02	0.03
**Day1_ QRS interval**	0.08±0.01	0.1±0.12	0.20
**Day2_ QRS interval**	0.09±0.11	0.09±0.02	0.79
**Day3_ QRS interval**	0.09±0.01	0.10±0.02	0.005
**Day1_QT interval**	0.38±0.03	0.38±0.02	0.36
**Day2_QT interval**	0.38±0.03	0.38±0.02	0.70
**Day3_QT interval**	0.39±0.03	0.39±0.02	0.89
**Day1_SBP**	120.26±11.05	118.17±10.62	0.33
**Day2_SBP**	123.98±10.80	124.66±13.55	0.75
**Day3_SBP**	125.64±15.34	127.57±16.58	0.54
**Day1_DBP**	67.12±7.09	67.37±7.48	0.86
**Day2_DBP**	72.49±7.72	73.00±10.06	0.77
**Day3_DBP**	73.55±9.05	76.14±11.32	0.20
**Day1_PR**	83.69±12.90	84.75±12.31	0.83
**Day2_PR**	87.82±10.49	89.95±12.33	0.35
**Day3_PR**	92.86±12.12	92.83±12.78	0.99
**Operation period**	242.30±48.73	233.10±48.50	0.26
**ACC_time**	38.00±7.77	36.40±10.37	0.70
**Pump_time**	61.80±25.72	48.80±15.36	0.18
**Day1_SrCr**	0.83±0.18	0.88±0.17	0.12
**Day2_SrCr**	0.84±0.20	0.86±0.19	0.39
**Day3_SrCr**	0.80±0.17	0.85±0.19	0.21
**Blood transfusion**	1.64±2.08	2.64±2.82	0.04
**ICU stay**	2.48±1.12	3.54±2.44	0.01
**Hospital stay**	5.64±1.48	6.90±2.88	0.004

ACC, aortic cross-clamp; DBP, diastolic blood pressure; ICU, intensive care unit; SBP, systolic blood pressure; SrCr, serum creatinine

**Table 7:** Multiple logistic regression analysis predicting the effect of crocin on atrial fibrillation, adjusted for sex, SBP and WBC

	**coefficient**	**SE**	**Wald**	**df**	**p-value**	**Odds ratio**
**Crocin**	-1.274	.533	5.765	1	.016	.266
**Sex (Female)**	0.357	.500	.560	1	.454	1.457
**SBP**	-0.003	.013	.027	1	.868	.998
**WBC**	0.000	.000	.875	1	.350	1.000
**Constant**	-1.150	2.059	.146	1	.702	-

## Discussion

In this double-blind, randomized, placebo-controlled trial, short-term prophylactic treatment with oral crocin from three days prior to the surgery and on the first three postoperative days, found to be significantly efficient on reducing development of POAF after CABG and heart valve replacement surgeries. Treatment with crocin significantly reduced oxidative stress (as shown by PAB serum levels), duration of ICU and hospital stay and the mean of blood transfusions during the operation period and ICU stay. ECG studies revealed that PR intervals were significantly longer during the three days after surgery in the crocin group. QRS interval durations but were significantly shorter on the third day after the surgery in comparison with the control group. 

Nevertheless, differences in ventricular arrhythmias, supraventricular arrhythmias (except AF), subgroup analysis of the arrhythmia incidence according to the operation type, CRP levels (as inflammation marker), QT intervals, and other intra-postoperative parameters were insignificantly different between the two groups. Moreover, we revealed that treatment with crocin was the only significant predictor of AF development. 

We assumed that crocin prevents POAF via antioxidant and anti-inflammatory properties. Oxidative stress is determined as an imbalance between the production of prooxidants and antioxidant defenses in favor of prooxidants (Nabatchican et al., 2014 ▶). The imbalance leads to increased ROS production and therefore, plays a major role in metabolic disorders and the pathogenesis of cardiovascular disease progress (Bierman, 1992 ▶; Ghazizadeh et al., 2020 ▶). In other words, ROS production leads to lipid peroxidation at the cell surface and tissue damage. Leukocytes activated by nicotinamide-adenine dinucleotide phosphate (NADPH) oxidase enzyme produce hydrogen peroxide. Then, lipid peroxidation, cell wall failure, and decreased mitochondrial activity lead to cell death and apoptosis (Elahi et al., 2008 ▶). Increased production of ROS can also cause changes in the number or activity of ion channels in the heart, including sodium and calcium channels and predispose the heart to AF (Barangi et al., 2018 ▶). Crocin has potent antioxidant and anti-inflammatory properties (Goli et al., 2012 ▶; Sefidgar et al., 2019 ▶). Based on some studies, consumption of crocin supplement decreases PAB level in subjects with metabolic syndrome (Nikbakht-Jam et al., 2016 ▶). In an animal study, it is demonstrated that crocin improves cardiac function after myocardial infarction. The antioxidant properties of crocin are mentioned effective in this process (Wang et al., 2018 ▶). It is shown that saffron established electrophysiological remodeling of the AV node in AF experimental model in rabbits (Khori et al., 2012 ▶). The hydroalcoholic extract of *C. sativus* increased the AV nodal refractoriness and zone of concealment. These depressant effects of *C. sativus* were suggested to be mediated with endogenous nitric oxide (NO) (Khori et al., 2012 ▶). Treatment with crocin significantly reduced oxidative stress in our study, which can be considered the main protective mechanism of crocin against POAF. 

The role of inflammation in the occurrence of AF was also determined. It is generally agreed that inflammation is involved in structural and electrophysiological processes in atrial remodeling and causes development and perpetuation of AF. However, it is still debatable if inflammation is sufficient to trigger AF as an initiating event. On the other hands, cause and effect relationship between inflammation and AF needs to be more understood (Galea et al., 2014 ▶; Del Campo et al., 2017 ▶; Meyre et al., 2020 ▶; Nomani et al., 2020 ▶). The effects of L-carnitine administration on POAF and CRP levels following CABG surgery were investigated in a randomized clinical trial. It was revealed that pretreatment with L-carnitine (3000 mg/d) inhibits and reduces the incidence of AF after CABG. Inflammation as measured by CRP was also reduced in the L-carnitine group compared with the control group, so researchers concluded that inflammation might be associated with the incidence of AF after surgery (Dastan et al., 2018 ▶). In another study, the effect of intravenous N-acetylcysteine (NAC) on the prevention of AF after CABG surgery was evaluated. It was demonstrated that perioperative treatment with intravenous NAC (50 mg/kg) can effectively reduce inﬂammation and the incidence of AF after CABG surgery (Soleimani et al., 2018 ▶). But perioperative supplementation with polyunsaturated omega-3 fatty acid (PUFA) did not decrease the risk of AF in the immediate postoperative period. PUFA also had no effect on the length of hospital stay, postoperative bleeding complications, or readmissions within one month after surgery (Joss et al., 2017 ▶). Effectiveness of corticosteroids in the prophylaxis of the POAF in patients undergoing elective CABG or valvular heart surgery was also assessed. It was concluded that treatment with 1 g methylprednisolone before surgery and then 100 mg hydrocortisone every 8 hr for the first 3 postoperative days is safe and effective in reducing the incidence of POAF in these patients. However, the intervention was not significantly effective on the length of hospital stay (Al-Shawabkeh et al., 2017 ▶). Notwithstanding the theory of the inflammatory mechanism in the development of POAF, the use of some anti-inflammatory drugs such as fish oil, unsaturated fatty acids, and colchicine has been ineffective in preventing this complication (Zakkar et al., 2015 ▶). The effects of saffron extract on serum anti-inflammatory and antioxidant variables in patients with type 2 diabetes mellitus were evaluated in another study. No improvement in homocysteine levels, antioxidant status or inflammatory biomarkers were shown (Shahbazian et al., 2019 ▶). Similarly, treatment with crocin was not effective on reducing CRP levels in our study. 

Due to the high prevalence and important outcomes of AF after surgery, research has been performed to prevent it. Conventional strategies for the prevention of POAF are based on modulation of the sympathetic nervous system, atrial conduction and atrial excitability time, in addition to the administration of β-blockers, digoxin, and amiodarone (Haghjoo, 2018 ▶). However, the noted strategies have limited efficacy and exert several adverse effects such as hypotension and bradycardia due to impaired neurohormonal and conduction activity. Therefore, development and progression of more efficacious and safe strategies are demanded to prevent POAF, yet. According to our research, crocin as a natural product seems to be an acceptable candidate for future research in this field. 

Some limitations should be mentioned for the present study. First, our results might be influenced by unknown variables, although we tried to omit confounding factors with homogenous randomization between the two groups. Second, in spite of recording the 12-lead ECG for assessment by two independent cardiologists blinded to the study in suspicious cases, there is a possibility of missing arrhythmia in ECG monitoring in the intensive care unit (ICU) and then in the coronary care unit (CCU). Third, we did not evaluate the effects of different doses of crocin on prevention of POAF. At last, the greatest limitation of this study was the small sample size and short duration of the intervention and follow-up. Further studies should be performed to investigate the efficacy and safety of the different doses of crocin in larger populations and during a longer period of time. 

Short-term prophylactic use of crocin both preoperatively and postoperatively seems to be effective in prevention of POAF in patients undergoing CABG or heart valve surgeries. It is concluded that crocin decreases POAF significantly, most probably through its antioxidant properties. However, further research with larger sample sizes and over a longer period of time are still warranted to examine the safety and impact of different doses of crocin, in addition to other possible advantages and mechanisms.

## Conflicts of interest

The authors have declared that there is no conflict of interest.
